# Efficacy of extracorporeal shock waves therapy for erectile dysfunction treatment: a systematic review and meta-analysis

**DOI:** 10.1186/s12610-025-00258-1

**Published:** 2025-03-17

**Authors:** Carla Juan-Casas, Raquel Leirós-Rodríguez, Ana González-Castro, Pablo Hernandez-Lucas

**Affiliations:** 1 Nursing and Physical Therapy Department, University of Leon, Astorga Ave, Ponferrada, 24401 Spain; 2 SALBIS Research Group, Nursing and Physical Therapy Department, University of Leon, Astorga Ave, Ponferrada, 24401 Spain; 3 Faculty of Physiotherapy, University of Vigo, Campus A Xunqueira, Pontevedra, 36005 Spain

**Keywords:** Electric stimulation therapy, Erectile dysfunction, Extracorporeal shock waves therapy, Genital diseases, Physiotherapy, Sexual disorders, Vasculogenic impotence, Thérapie de Stimulation électrique, Dysfonction érectile, Thérapie par Ondes de Choc extracorporelles, Maladies génitales, Physiothérapie, Troubles sexuels, Impuissance vasculogénique

## Abstract

**Background:**

Erectile dysfunction is becoming a public health problem, affecting 22% of men over 40 years of age, where one of the first lines of treatment for this pathology is the use of drugs, so it is necessary to know the effectiveness of new non-invasive alternative therapies that limit the consumption of these substances in the general population. Therefore, the aim of this study was to evaluate the efficacy of extracorporeal shock waves therapy for the treatment of erectile dysfunction. To achieve this, a systematic review was carried out through the databases PubMed, Scopus, Science Direct, Cinhal, Medline, and Web of Science; using the search terms, Erectile Dysfunction, Physical Therapy Modalities, Physical Therapy Specialty, Rehabilitation and Shock Wave Therapy.

**Results:**

The search ended with a total of 15 articles, differentiating between two study groups, those patients suffering from organic erectile dysfunction (*n* = 12) and those suffering from the same pathology after undergoing radical prostatectomy with nerve sparing (*n* = 3). The combined analysis showed that the group treated with extracorporeal shock waves therapy had a significant increase in erectile function compared to the controls. The Difference in Means was 2.96 points (95% CI: 1.93 to 4.61; *p* < 0.001; I^2^ = 63.45).

**Conclusions:**

Extracorporeal shock waves therapy appears to have a positive effect in the treatment of erectile dysfunction, with these changes being reflected in different variables such as erectile function, erectile efficacy or sexual satisfaction. Its efficacy seems to increase with interventions that include two weekly sessions and with the application at least 6000 pulses in each session.

**Trial registration:**

PROSPERO Registration code: CRD42021230001.

## Résumé

**Contexte:**

La dysfonction érectile est un problème de santé publique, affectant 22% des hommes de plus de 40 ans; l’une des premières lignes de traitement en est l’utilisation de médicaments. Il est donc nécessaire de connaître l’efficacité des nouvelles thérapies alternatives non invasives qui limitent l’utilisation de ces substances dans la population générale. Aussi, le but de cette étude était d’évaluer l’efficacité de la thérapie par ondes de choc extracorporelles dans le traitement de la dysfonction érectile. Pour ce faire, une revue systématique a été réalisée à l’aide des bases de données PubMed, Scopus, Science Direct, Cinhal, Medline et Web of Science, en utilisant comme termes de recherche, Dysfonction érectile, Modalités de physiothérapie, Spécialité de physiothérapie, Réadaptation et Thérapie par ondes de choc.

**Résultats:**

La recherche a retenu un total de 15 articles, différenciant deux groupes d’étude, les patients souffrant de dysfonction érectile organique (*n* = 12) et ceux souffrant de la même pathologie après avoir subi une prostatectomie radicale avec conservation des nerfs (*n* = 3). L’analyse combinée a montré que le groupe traité par thérapie par ondes de choc extracorporelles présentait une augmentation significative de la fonction érectile par rapport aux témoins. La différence de Moyennes était de 2,96 points (IC à 95%: 1,93 à 4,61; *p* < 0,001; I2 = 63,45).

**Conclusions:**

La thérapie par ondes de choc extracorporelles semble avoir un effet positif dans le traitement de la dysfonction érectile, ces changements se reflétant dans différentes variables telles que la fonction érectile, l’efficacité érectile ou la satisfaction sexuelle. Son efficacité semble augmenter avec des interventions qui comprennent deux séances hebdomadaires et avec l’application d’au moins 6000 impulsions dans chaque séance.

**Enregistrement d'essai:**

Code d’enregistrement PROSPERO: CRD42021230001.

## Introduction

Erectile dysfunction (ED) is known as the inability to maintain an erection sufficient to achieve penetration and, thus, satisfactory sexual intercourse [1]. Its origin can be psychogenic, nervous, endocrine or vascular, and it is often associated with the presence of certain risk factors such as type II diabetes mellitus, arterial hypertension, metabolic syndrome, depression, toxic habits, obesity and sedentary lifestyle [2]. The prevalence of ED increases with age [3]. ED is becoming a public health problem due to the aging of the population, establishing that, in 2025, 322 million men will suffer from ED worldwide [2, 3].

This pathology has a great impact on the quality of life of patients, as well as on their self-esteem, causing anxiety and depression. It is not only a physiological dysfunction at the sexual level, but it also interferes at the social and emotional level, causing a detriment to the patient's health [4, 5].

The treatment should be personalized for each patient based on the invasiveness, tolerability, and efficacy of the different therapeutic options, as well as the patient's needs and expectations. In this context, patients should receive comprehensive counseling regarding all available treatment modalities: intracavernosal injection, vacuum device, oral therapy with hosphodiesterase type five inhibitor drug (PDEI-5), intraurethral or topical alprostadil and physical therapy treatments [6]. The PDEI-5 achieves penile erection by relaxing the smooth musculature of the corpora cavernosa, treating the symptoms without affecting the pathophysiological mechanism [7]. The intracavernous injection of vasoactive drugs, which achieve the final objective in a short time, and without the need for sexual desire on the part of the patient [8]. As a non-pharmacological alternative, there are vacuum erection devices, lifestyle changes to minimize risk factors and specialized physiotherapy treatment (electrostimulation, therapeutic exercise, pelvic floor exercises, electromyographic biofeedback, manual therapy and health education) [9].

Particularly, physical therapy treatments act, among others, on the musculature of the perineum, which plays a great role in sexual functionality. Specifically, the bulbospongiosus and ischiocavernosus muscles are responsible for facilitating penile erection, raising intracavernosal and intraspongiosal pressure, also contributing to ejaculation [10, 11]. Therefore, this physiotherapy can improve the sexual health of men with ED by being a means of comprehensive evaluation and treatment of sexual dysfunctions of musculoskeletal origin and by influencing the various risk factors for their prevention [11]. One of the main treatments for ED is rehabilitation of the musculature through exercise or electrostimulation, achieving greater awareness and motor control [10].

Among the existing methods and techniques, extracorporeal shock waves therapy (ESWT) stands out: this is defined as a disturbance in pressure, propagating rapidly through a medium, generally water or through the application of a gel on the head of the machine to facilitate penetration into the tissues. As energy penetrates the medium, it causes an increase in tension in the area, as well as a cavitation phenomenon [12]. At the biological level, ESWT alters the permeability of neuronal membranes, increasing the action potential and, consequently, achieving an analgesic effect. Furthermore, they also increase blood flow in the area to be treated, improving the healing processes mediated by inflammation [12, 13].

In addition, ESWT can regenerate blood vessels and neuronal tissue, improving erectile function [14]. All this is achieved by increasing penile perfusion and improving the neurophysiology involved in erection [15]. However, this is a therapeutic option that is not widely used and has not been protocolized, and the most appropriate application parameters for the treatment of ED have not been established. Therefore, a systematic review was considered necessary to determine the efficacy of ESWT for the treatment of ED and to identify the most appropriate application parameters for the treatment of these patients.

## Materials and methods

This study was prospectively registered on PROSPERO (ID: CRD42021230001) and followed the Preferred Reporting Items for Systematic Reviews and Meta-analyses (PRISMA) in Exercise, Rehabilitation, Sport medicine and Sports Science reporting guidelines and the recommendations from the Cochrane Collaboration [16, 17]. The PICO question was then chosen as follows: P – population: men with ED; I – intervention: ESWT; C – control: placebo and/or pharmacological treatment; O – outcome: erectile function; S – study designs: experimental studies.

A systematic search of publications was conducted in August 2024 in the following databases: PubMed, Scopus, Science Direct, Cinhal, Medline, and Web of Science. The search strategy included different combinations with the following Medical Subject Headings (MeSH) terms: *Erectile dysfunction, Physical therapy modalities, Physical therapy speciality, Rehabilitation,* and *Shock wave therapy*. The search strategy according to the focused PICOS question is presented in Table 1.

**Table 1:** Search strategy according to the focused question (PICO)

Database	Search equation
PubMed	(Erectile Dysfunction[MeSH Terms]) AND (Physical Therapy Modalities[MeSH Terms]) (Erectile Dysfunction[MeSH Terms]) AND (Physical Therapy Speciality[MeSH Terms]) (Erectile Dysfunction[MeSH Terms]) AND (Rehabilitation medicine[MeSH Terms]) (Erectile Dysfunction[MeSH Terms]) AND (Rehabilitation[MeSH Terms]) (Erectile Dysfunction[MeSH Terms]) AND (Shock wave therapy[MeSH Terms])
ScienceDirect	("Erectile Dysfunction" [Mesh]) AND ("Physical Therapy Modalities” [Mesh]) ("Erectile Dysfunction" [Mesh]) AND ("Physical Therapy Speciality” [Mesh]) ("Erectile Dysfunction" [Mesh]) AND ("Rehabilitation medicine “ [Mesh]) ("Erectile Dysfunction" [Mesh]) AND ("Rehabilitation “ [Mesh]) ("Erectile Dysfunction" [Mesh]) AND ("Shock wave therapy “ [Mesh])
Cinahl	(MH "Erectile Dysfunction") AND (MH "Physical Therapy Modalities”) (MH "Erectile Dysfunction") AND (MH "Physical Therapy Speciality”) (MH "Erectile Dysfunction") AND (MH "Rehabilitation medicine “) (MH "Erectile Dysfunction") AND (MH "Rehabilitation “) (MH "Erectile Dysfunction") AND (MH "Shock wave therapy “)
Medline	(MH "Erectile Dysfunction") AND (MH "Physical Therapy Modalities”) (MH "Erectile Dysfunction") AND (MH "Physical Therapy Speciality”) (MH "Erectile Dysfunction") AND (MH "Rehabilitation medicine “) (MH "Erectile Dysfunction") AND (MH "Rehabilitation “) (MH "Erectile Dysfunction") AND (MH "Shock wave therapy “)
Web of Science	("Erectile Dysfunction" [Mesh]) AND ("Physical Therapy Modalities” [Mesh]) ("Erectile Dysfunction" [Mesh]) AND ("Physical Therapy Speciality” [Mesh]) ("Erectile Dysfunction" [Mesh]) AND ("Rehabilitation medicine “ [Mesh]) ("Erectile Dysfunction" [Mesh]) AND ("Rehabilitation “ [Mesh]) ("Erectile Dysfunction" [Mesh]) AND ("Shock wave therapy “ [Mesh])
Scopus	("Erectile Dysfunction" [Mesh]) AND ("Physical Therapy Modalities” [Mesh]) ("Erectile Dysfunction" [Mesh]) AND ("Physical Therapy Speciality” [Mesh]) ("Erectile Dysfunction" [Mesh]) AND ("Rehabilitation medicine “ [Mesh]) ("Erectile Dysfunction" [Mesh]) AND ("Rehabilitation “ [Mesh]) ("Erectile Dysfunction" [Mesh]) AND ("Shock wave therapy “ [Mesh])

### Study selection

After removing duplicates, two reviewers (PT. C. J.-C. and PhD. R. L.-R.) independently screened articles for eligibility. In case of disagreement, both reviewers debated until an agreement was reached. For the selection of results, the inclusion criteria established that: (a) the study had to be experimental; (b) the intervention had to include ESWT; (c) the sample had to consist of men with ED; and (d) if a control group was included, it had to receive either a placebo and/or pharmacological treatment. On the other hand, studies were excluded from this review if: (a) they employed a non-experimental methodology (e.g., reviews, meta-analyses, or editorials); or (b) if their full text was not available.

After screening the data, extracting, obtaining and screening the titles and abstracts for inclusion criteria, the selected abstracts were obtained in full texts. Titles and abstracts lacking sufficient information regarding inclusion criteria were also obtained as full texts. Full text articles were selected in case of compliance with inclusion criteria by the two reviewers using a data extraction form. The two reviewers mentioned independently extracted data from included studies using a customized data extraction table in Microsoft Excel.

The data extracted from the included articles for further analysis were: title, authors, journal and year, characteristics of the sample (age, inclusion and exclusion criteria, and number of participants), study-specific parameters (study type, duration of the intervention, techniques applied), ESWT application parameters (frequency, application area, number of pulses and devices used) and results obtained (variables analyzed, instruments used and time of follow-up). Tables were used to describe both the studies’ characteristics and the extracted data.

### Assessment of risk of bias

The ROBINS-I tool was used to assess the risk of bias in non-randomized studies [18], while the RoB tool was used to assess the risk of bias in randomized studies [19]. Additionally, the Grades of Recommendations Assessment, Development, and Evaluation (GRADE) approach was employed to assess the quality of the evidence when conducting meta-analysis [20].

### Statistical analysis

A meta-analysis was conducted to synthesize the results of the included studies, using the Difference in Means (DM) as the effect measure for erectile function between the experimental group and the control group in each study.

The DM was calculated by subtracting the mean of the control group from the mean of the experimental group for each study. DM were interpreted using the following cut-off values: 0 to 0.2: very small; from 0.2 to 0.5: small; from 0.5 to 0.8: moderate; and from 0.8: strong [21]. Heterogeneity among the studies was assessed using the I^2^ statistic and the p-value associated with Cochran’s Q test [22]. Significant heterogeneity was detected (I^2^ > 50% or *p* < 0.1), so a random-effects model based on the DerSimonian and Laird method was used to adjust for differences between the studies. The results are presented with 95% confidence intervals (95% CI) to reflect the precision of the combined estimate of the DM. A positive DM value indicates a higher mean in the experimental group compared to the control group, while a negative value indicates a higher mean in the control group. The statistical analysis was performed using Comprehensive Meta-Analysis (CMA) V2 software (Biostat, NJ).

## Results

### Study selection

**Fig. 1:**
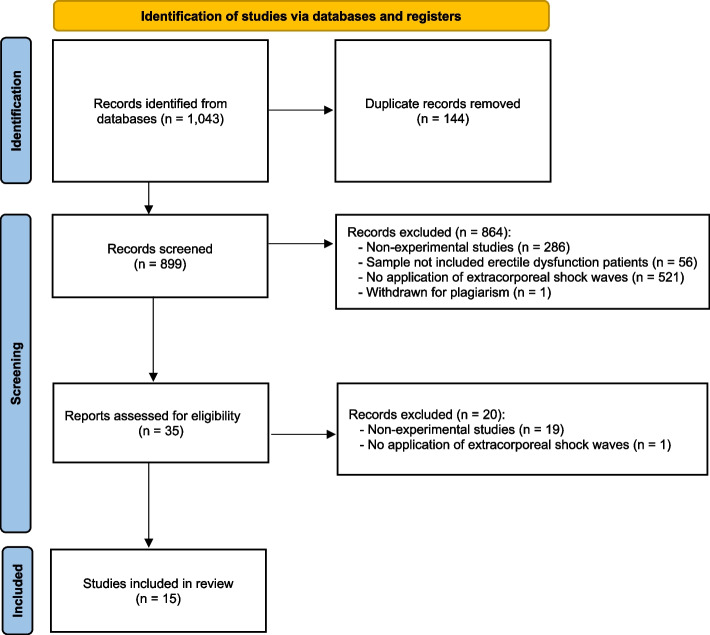
Preferred Reporting Items for Systematic Reviews and Meta-Analyses (PRISMA) flow diagram

Out of 1,043 search results, 899 studies were considered eligible for inclusion after removing duplicates. Among the 899 papers screened, 864 were excluded after abstract and title screening. After the first reading of all candidates’ full texts, Kappa score of reviewers 1 and 2 was 0.96, indicating a very high agreement. Of the 35 full-text articles assessed for eligibility, 15 were finally included in the synthesis, as depicted by the PRISMA flowchart in Fig. 1. All the data necessary for analysis was obtained from all the studies analyzed.

### Study characteristics and risk of bias

Regarding the study population, ten investigations analyzed treatment success in patients with organic erectile dysfunction (ED) [24–35], while the studies by Baccaglini et al. [36] and Zewin et al. [37] evaluated the effectiveness of ESWT in patients who underwent total or partial prostatectomy with nerve sparing. As shown in Table 2, the level of evidence provided was predominantly I (83.3%) [23–33, 36, 37], while 16.7% corresponded to level IV evidence [34, 35].

**Table 2:** Methodological characteristics of the studies analyzed

**Authors**	**Design**	**Sample size**	**Intervention**		**Number of sessions** **(Frequency)**	***P***	**D** (mm)	**F **(Hz)	**I ** (mJ/mm^2^)	**LE**
			**Experimental group**	**Control group**						
Olsen et al. (2014) [24]	RCT	105	ESWT	Placebo ESWT + applicator	5 (1 session/week)	3000	*---*	5	0.15	I
Yee et al. (2014) [33]	RCT	58	ESWT	Placebo ESWT	12 (2 sessions/week)	1500	18	2	0.09	I
Kitrey et al. (2016) [32]	RCT	55	ESWT + PDEI-5	Placebo ESWT	12 (2 sessions/week)	1500	*---*	2	0.09	I
Fojecki et al. (2017) [26]	RCT	118	ESWT	Placebo ESWT	10 (1 session/week)	600	10	5	0.09	I
Qi et al. (2017) [35]	QES	60	Group 1: ESWT Group 2: Vacuum erection device	---	8 (2 sessions/week)	1500	*---*	2	*---*	IV
Sramkova et al. (2017) [27]	RCT	60	ESWT	Placebo ESWT + gel	4 (2 sessions/week)	6000	15	*---*	0.16	I
Kalyvianakis et al. (2018) [34]	QES	Phase 1: 42 Phase 2: 36	ESWT	---	Phase 1: 6/12 (1-2 sessions/week) Phase 2: 6 (2-1 sessions/week)	5000	*---*	8	0.05	IV
Zewin et al. (2018) [37]	RCT	128	ESWT	PDEI-5	12 (2 sessions/week)	1500	*---*	2	0.09	I
Baccaglini et al. (2019) [36]	RCT	77	ESWT + PDEI-5	PDEI-5	8 (1 session/week)	2400	40	5	0.09	I
Vinay (2019) [28]	RCT	76	ESWT	ESWT + bandage	4 (1 session/week)	5000	*---*	*---*	0.09	I
Kim et al. (2020) [31]	RCT	81	ESWT	Placebo ESWT	12 (2 sessions/week)	3000	*---*	5	12-20	I
Geyik (2021) [23]	RCT	184	ESWT	ESWT + PRP	5 (1 session/week)	1800	70	*---*	0.09	I
Ortac et al. (2021) [29]	RCT	66	ESWT	Placebo	4 (1 session/week)	3000	30	5	0.20	I
Shendy et al. (2021) [25]	RCT	42	ESWT + PFMT	Placebo ESWT + PFMT	12 (2 sessions/week)	3000	*---*	*---*	0.09	I
Motil et al. (2022) [30]	RCT	32	ESWT	Placebo	4 (1 session/week)	4000	10	8	0.16	I

*P* Pulses per session, *D* Depth of the application, *F* Frequency of the application, *LE* Level of evidence, *QES* Quasi-Experimental study, *CS* Controlled study, *RCT* Randomized controlled trial, *QES* Quasi-experimental study, *ESWT* Extracorporeal shock waves therapy, *PDEI-5* Phosphodiesterase type five inhibitor drug, *PFMT* Pelvic Floor Muscle Training, –- Not described

Of the 15 articles analyzed, 13 were randomized controlled trials in which the control group received: (a) a PDEI-5 drug [23, 36, 37]; (b) simulated ESWT with an applicator that blocked the passage of waves [24, 25]; (c) a gel or dressings that prevented energy transmission [26–30]; or (d) a zero energy set on the device [31–33].

The remaining two investigations followed a quasi-experimental methodology, comparing the efficacy of different treatment protocols based on the number of sessions [34] and the use of vacuum erection devices [35]. The methodological characteristics of the investigations are shown in Table 2. and a summary of the findings of each can be found in Table 3.

**Table 3:** Characteristics and results of the studies analyzed

Authors	Inclusion criteria	Exclusion criteria	Results identified
Olsen et al. (2014) [24]	Age between 18 and 80 years old. Diagnosis of ED of more than 6 months of evolution. Maintenance of stable sexual partner of at least 3 months of evolution. Erection Hardness Scale score of less than 2 points. International Index of Erectile Function score of less than 20 points	Diagnosis of psychogenic ED, neurological and/or cardiac pathology that prevents sexual intercourse. History of prostatectomy, rectal removal, pelvic radiotherapy and/or cancer in the previous five years. Treatment with antiandrogens	Improved erection quality No improvement in erection function
Yee et al. (2014) [33]	Age over 18 years old. Diagnosis of ED of more than 6 months of evolution. Maintenance of a heterosexual couple of at least 6 months. Men's Sexual Health Inventory score lower than 21 points	Diagnosis of non-vascular endocrine, neurological, pharmacological or penile deformities. History of pelvic surgery and/or pelvic radiotherapy treatment. Presence of penile implant	Improvement of erectile function in patients with severe ED No improvement in erection quality
Kitrey et al. (2016) [32]	Initial positive response to PDEI-5 and termination of treatment due to lack of efficacy in the last year. Erection Hardness Scale score of at least 2 points	Presence of penile malformations and/or unstable medical situation. Diagnosis of neurological and/or hormonal pathologies. History of prostate cancer	Improvement of erectile function, erection quality, penile hemodynamics
Fojecki et al. (2017) [26]	Age over 40 years old. Diagnosis of ED of more than 6 months of evolution. Maintenance of stable relationship of at least 3 months	History of surgery, pelvic radiotherapy and/or use of penile prosthesis. Treatment with anticoagulants and/or antiandrogens. Presence of penile deformities, and/or testosterone levels below 8 nmol/dL. Diagnosis of severe cardiac and/or pulmonary pathologies and/or neurological and/or psychiatric disorders. International Index of Erectile Function score higher than 25 points. Existence of pregnant partner	Improvement of quality of sexual life No improvement in erectile function and erection quality
Qi et al. (2017) [35]	Age between 20 and 55 years old. Diagnosis of ED according to the European and Chinese ED Guidelines. Presence of abnormal penile tumescence and rigidity. International Index of Erectile Function score less than 22 points	History of prostate surgery, trauma and/or cancer in the previous 5 years. Diagnosis of Diabetes Mellitus, arterial hypertension, spinal cord injury, psychiatric pathology and/or hematological diseases with clinical manifestations. Presence of penile deformities. Consumption of androgens or antiandrogens. History of previous treatment with medication, vacuum erection devices, intracavitary cavernous injection and/or intraurethral pharmacotherapy	Improved erectile function, sexual satisfaction, erection quality and ability to maintain sexual intercourse
Sramkova et al. (2017) [27]	Diagnosis of ED of less than 6 months of evolution. Maintenance of stable partner and regular sexual activity with, at least, two relations per week	History of pelvic surgery. Diagnosis of psychogenic or neurological ED and/or neurological pathology	Improvement of erectile function, erection quality, ability to maintain sexual intercourse and sexual satisfaction of the patient and partner
Kalyvianakis et al. (2018) [34]	Cavernous artery peak systolic velocity less than 35 cm/s. International Index of Erectile Function score less than 26 points	Diagnosis of psychiatric pathologies, psychogenic or neurological ED, active cancer, untreated endocrine diseases, uncontrolled Diabetes Mellitus, arterial hypertension, cardiovascular pathology and/or hemophilia. Previous treatment with PDEI-5. Presence of penile deformities and/or high risk of thrombosis. History of penile and/or pelvic surgery	Improvement of erectile function, sexual satisfaction, penile hemodynamics
Zewin et al. (2018) [37]	Diagnosis of bladder cancer with muscle invasion. Presence of sexual motivation. Maintenance of stable relationship of more than 6 months of antiquity. Sexually active men without erectogenic aids before the cancer intervention	Diagnosis of Peyronie's disorder, Diabetes Mellitus and/or psychiatric disorders. Presence of inflammation in the area of application and/or postoperative complications	Improvement of erectile function, erection quality and hemodynamics of the cavernous arteries
Baccaglini et al. (2019) [36]	Age over 75 years old. Maintenance of a heterosexual relationship of at least 3 months. History of prostatectomy with nerve preservation. International Index of Erectile Function score less than 18 points	Previous treatment with pelvic radiotherapy and/or antiandrogens. Diagnosis of psychiatric pathologies, hypogonadism and/or uncontrolled diabetes mellitus. Presence of postoperative complications	Improvement of erectile function and urinary continence
Vinay (2019) [28]	Age over 18 years old. Diagnosis of ED of vascular origin without response to PDEI-5 of between 6 months and 7 years of evolution. International Index of Erectile Function score lower than 26 points	History of treatment with PDEI-5 and/or pelvic radiotherapy and/or cancer in the year prior to the study. Diagnosis of non-vascular ED and/or acute or chronic disease. Treatment with psychotropic drugs. Presence of penile deformities and/or a value greater than 3 on the International Normalized Ratio	Improvement of erectile function, erection quality, sexual satisfaction and ability to maintain sexual intercourse
Kim et al. (2020) [31]	Age over 20 years old. Diagnosis of medium or moderate ED of at least 6 months of evolution. Maintenance of stable sexual relationship for more than 3 months	Diagnosis of severe and/or psychogenic ED, neurological and/or cardiac pathologies that inhibit sexual contact. History of prostatectomy, rectal excision and/or pelvic radiotherapy. Presence of anatomical malformations. Treatment with anticoagulants	Improvement of erectile function, erection quality and sexual satisfaction
Geyik (2021) [23]	Patients who used 5 mg daily of PDEI-5 and still could not achieve a penile erection that would allow satisfactory sexual intercourse	Glycosylated hemoglobin levels > 7 ng/ml. Hypogonadism. Cardiac and antihypertensive medications not adjusted in consultation. History of pelvic surgery. History of degenerative neurological disorders. Lack of information	Significant improvements in sexual function in both groups. Improvements in intravaginal ejaculation latency time in Group 2
Ortac et al. (2021) [29]	Age between 18 and 74 years. Diagnosis of mild ED and confirmed with Doppler ultrasound (International Index of Erectile Function score = 17–25)	Uncontrolled diabetes. Testosterone deficiency. Erection drugs during the first 4 weeks of study. Previous treatment with PDEI-5. History of concomitant neurological, hematological, cardiovascular disease or cancer	Improvement of erectile function
Shendy et al. (2021) [25]	Age over 18 years old. Body Mass Index less than 30 kg/m^2^. Diagnosis of controlled type II diabetes mellitus and medium or moderate ED of at least 6 months of evolution	History of pelvic surgery. Diagnosis of chronic psychiatric, neurological and/or hematological pathologies. Presence of penile deformities. Non-response to PDEI-5 and/or consumption in the month prior to the study	Improved erectile function and penile hemodynamics
Motil et al. (2022) [30]	Patients after surgery who did not have ED preoperatively and suffered mild-severe postoperative ED	Previous surgery or radiotherapy to the pelvic region. Anatomical abnormalities of the penis. Chronic hematologic disease. Oral or injectable anti-androgens. Cardiovascular conditions that impede sexual function	Improvement of erectile function

*ED* Erectile dysfunction, *PDEI-5* Phosphodiesterase type 5 inhibitor drug

### Application parameters

Regarding the application of ESWT, a great disparity was identified in terms of the duration of the treatments. This varied between 2 [31] and 36 weeks [34], with protocols of 9 weeks being the most frequent [25–27, 33, 37]. The disparity in the duration of the protocols is mainly due to the design of the interventions in one [23, 28–32, 35, 36] or two treatment cycles [23–27, 33, 34, 37]. Those investigations in which the intervention was divided into two treatment cycles mostly scheduled the intervention in three-week periods with three weeks of rest in between [25–27, 33, 37].

Similarly, the frequency of sessions also varied between one [23, 24, 28–30, 32, 36] and two weekly sessions [25–27, 31, 33–35, 37]. Kalyvianakis et al. [34] compared the efficacy of both frequencies of application (finding no difference between the two options in erectile function, although they did find a difference in the patients' perception of their sexual satisfaction, which was higher when two weekly sessions were applied).

The total dose of ESWT applied varied between 1500 [28] and 54,000 pulses [26]. Furthermore, the dose per session applied varied between 30,029 and 6000 pulses [31]; the most frequent application was 1500 pulses per session [27, 33, 35, 37].

Regarding the area of application, this was in the crura and penile shaft in most of the studies [23, 26, 28, 29, 33, 35, 36]. In addition, another investigation added the penile hilum to these two locations [34]. The other most frequent application area was the corpora cavernosa in isolation [26, 30] or in combination with the penile crura [31, 32]. Finally, one investigation applied ESWT to the base and shaft of the penis and the area most proximal to the glans penis [25]. Zewin et al. [37] did not provide information on the location of the application.

In addition, two investigations combined the application of ESWT with other treatments: Shendy et al. [25] included the performance of perineal muscle exercises with the Kegel protocol (Pelvic Floor Muscle Training) three days a week and Baccaglini et al. [36] added the administration of PDEI-5.

### Results of the analyzed studies

All the analyzed studies included the evaluation of erectile function through the International Index of Erectile Function, obtaining significantly better results with the application of ESWT than with its simulated application [24, 25, 27–32], except in the study by Fojecki et al. [26]. Furthermore, ESWT showed improvements like those achieved with vacuum devices [35] and PDEI-5 consumption [36, 37], and the investigation by Yee et al. [33] only reported improvements with ESWT application in those patients with severe ED [33]. Finally, this variable improved similarly in the study by Kalyvianakis et al. [34] regardless of the number of ESWT sessions received.

Erection quality was evaluated in all cases by the Erection Hardness Score with positive results on most occasions [26–29]. Neither Fojecki et al. [26] nor Olsen et al. [24] identified superior results with the real application of ESWT than with the simulated application of ESWT, and Yee et al. [33] did not identify changes in this variable. In addition, the improvements in erection quality shown by ESWT were like those achieved with vacuum erection devices [34] and PDEI-5 administration [37].

Sexual satisfaction was assessed in all cases through the Sexual Encounter Profile. This variable showed significantly superior results after the application of ESWT in two investigations [27, 31], although its correct application did not cause superior changes compared to the use of vacuum erection devices [35]. Kalyvianakis et al. [34] identified statistically superior changes with the application of two sessions per week compared to the weekly application of this intervention. Finally, Vinay [28] failed to modify this variable after their intervention.

Penile blood flow and perfusion were assessed by veno-occlusive plethysmography [32] and Doppler ultrasound [24, 34, 37]. In all four studies, significant changes in the records were achieved, although these were not superior to those achieved with PDEI-5 administration [37].

The ability to maintain sexual intercourse was quantified by the Global Assessment Question [27, 28, 35]. Again, in the three studies, significant changes were identified with the application of ESWT, although these were not greater than those achieved with the use of vacuum erection devices [35].

The degree of satisfaction with the treatment received was assessed by means of the Erectile Dysfunction Inventory of Treatment Satisfaction [26], the Clinical Global Impression Scale [32] and a direct question formulated ad hoc [27]. It was only with this last method that significant improvements were identified with the application of ESWT. In fact, Kitrey et al. [32] did not identify differences between the study groups, and Fojecki et al. [26] identified low levels of satisfaction in the experimental and control groups.

The quality of sexual life was assessed only twice using the Sexual Quality of Life-Men [26, 28]. Both found disparate results: Vinay [28] reported significantly higher results with the application of ESWT, and the data obtained by Fojecki et al. [26] were like the baseline data after the intervention.

Finally, only one study considered partner satisfaction [27]. The authors found significantly superior results in this variable after the application of ESWT when analyzing the data obtained by a direct question formulated ad hoc*.*

### Results of the meta-analysis on erectile function

A total of 10 studies were included in the meta-analysis [25–27, 29–33, 36, 37] that evaluated the effectiveness of ESWT therapy compared to simulated ESWT or PDEI-5 controls in improving erectile function. The primary outcome measure was the DM in the Index of Erectile Function between the ESWT group and the control groups.

The combined analysis showed that the group treated with ESWT had a significant increase in erectile function compared to the controls. The DM was 2.96 points (95% CI: 1.93 to 4.61; *p* < 0.001; I^2^ = 63.45).

**Fig. 2:**
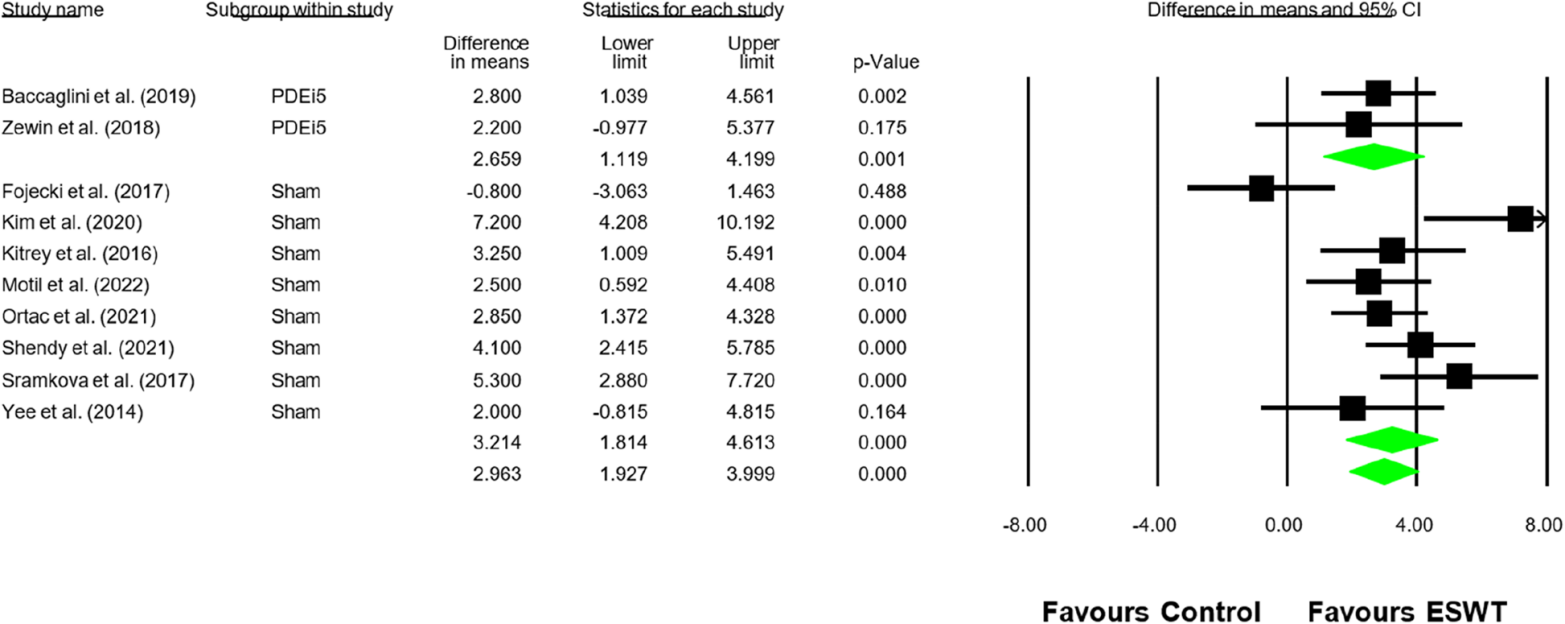
Forest plot for erectile function

In the subgroup analysis, the comparison between ESWT and the simulated ESWT group showed a DM of 3.21 points (95% CI: 1.81 to 4.61; *p* < 0.001), indicating that patients treated with ESWT experienced a significantly greater increase in erectile function compared to those in the simulated ESWT group (Fig. 2).

On the other hand, the comparison between ESWT and the group treated with PDEI-5 showed a DM of 2.66 points (95% CI: 1.20 to 4.20; *p* = 0.001), suggesting a significant increase in erectile function in the ESWT group compared to standard treatment with PDEI-5.

### Risk of bias for individual studies

The risk of bias within individual studies was determined to be critical in ten studies (66,7%) [27–31, 33–37] while five studies had a low risk of bias [23–26, 32] (Table 4). Additionally, the certainty of the evidence obtained was assessed as low for the variable of erectile function (Table 5).

**Table 4:** Risk of bias for included studies

ROBINS-I tool results for non-randomized studies								
**Authors**	**Confounding**^**a**^	**Selection**^**b**^	**Classification of interventions**	**Derivation from intended intervention**	**Missing data**^**c**^	**Outcomes**	**Selective reporting**^**d**^	**Overall**
Qi et al. (2017) [35]	Critical	Low	Low	Low	Low	Low	Critical	**Critical**
Kalyvianakis et al. (2018) [34]	Critical	Low	Low	Low	Low	Low	Low	**Critical**
**RoB tool results for randomized studies**								
**Authors**	**Random sequence (selection bias)**	**Allocation concealment (selection bias)**	**Blinding of participants and personnel (performance bias)**	**Blinding of outcome assessment (detection bias)**	**Incomplete outcome data (attrition bias)**	**Selective reporting (reporting bias)**	**Other bias**	**Overall**
Olsen et al. (2014) [24]	Low	Low	Low	Low	Low	Low	Low	**Low**
Yee et al. (2014) [33]	Low	Low	Low	Low	High	Low	Low	**High**
Kitrey et al. (2016) [32]	Low	Low	Low	Low	Low	Low	Low	**Low**
Fojecki et al. (2017) [26]	Low	Low	Low	Low	Low	Low	Low	**Low**
Sramkova et al. (2017) [27]	Low	Low	Low	High	High	Low	Low	**High**
Zewin et al. (2018) [37]	Low	Low	High	High	High	Low	Low	**High**
Baccaglini et al. (2019) [36]	Low	Low	High	High	High	Low	Low	**High**
Vinay (2019) [28]	Low	Low	Low	High	Low	Low	Low	**High**
Kim et al. (2020) [31]	Low	Low	Low	Low	High	Low	Low	**High**
Geyik (2021) [23]	Low	Low	Low	Low	Low	High	Low	**High**
Ortac et al. (2021) [29]	Low	Low	Low	Low	High	Low	Low	**High**
Shendy et al. (2021) [25]	Low	Low	Low	Low	Low	Low	Low	**Low**
Motil et al. (2022) [30]	Low	Low	Low	Low	Low	Low	Low	**Low**

^a^Risk of bias from confounding was considered critical when confounding was not inherently controlled for (i.e. no or limited adjustment)

^b^Selection bias was critical when selection into the study was very strongly related to intervention and outcome. This occurred when the study included men with diagnoses other than erectile dysfunction

^c^Risk of bias due to missing data was considered moderate when there appeared to be a substantial amount of missing data. In these cases, the proportions of and reasons for missing data might differ across interventions groups. Of note, the majority of studies did not report on missing data. The risk of bias for these were classified as low but could also be considered “unknown”

^d^The studies with a moderate risk for selective outcome reporting were those that did not provided a pre-registered protocol

**Table 5:** Certainty of the evidence (GRADE)

**Outcomes**	**Number of participants** **(studies)**	**Risk of bias**^**a**^	**Inconsistency**^**b**^	**Indirectness**	**Imprecision**	**Other considerations**	**Certainty of the evidence** **(GRADE)**	
Erectile function	654(10 RCTs)	Low	Very low	Moderate	Low	None	⨁⨁◯◯Low	

*RCT* Randomized clinical trial; SMD: standardized mean difference

^a^The average risk of bias of the studies according to the ROBINS-I and RoB tools

^b^Low methodological heterogeneity but high statistical heterogeneity among trials (I^2^> 25%)

### Risk of publication bias

**Fig. 3:**
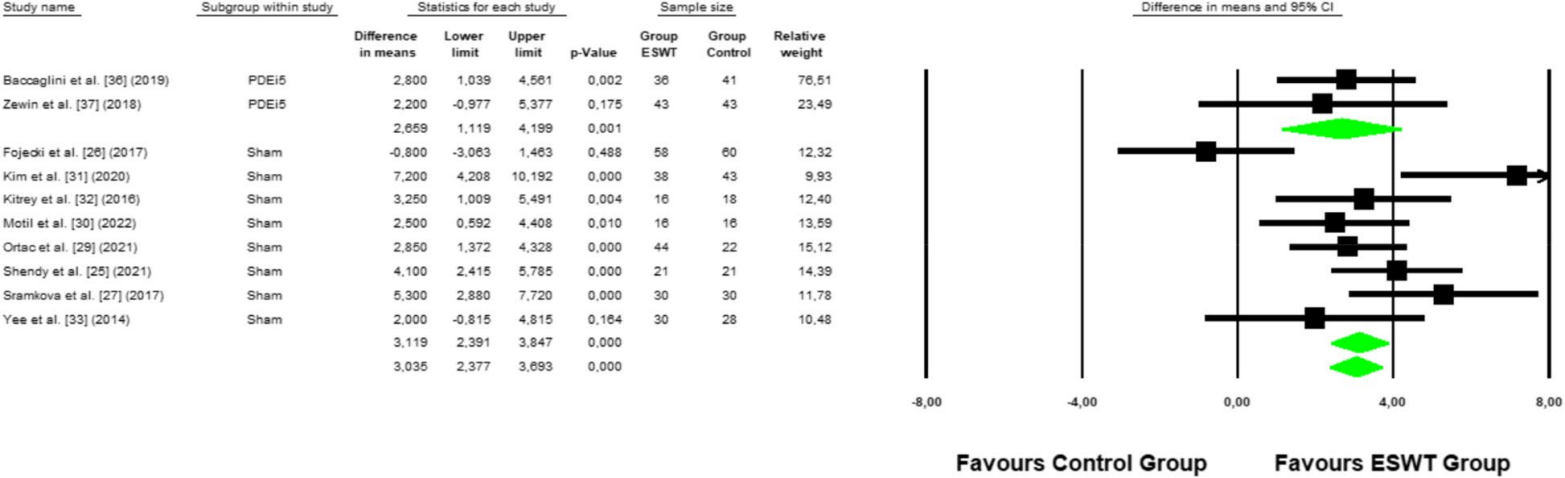
Funnel plot for erectile function

Egger’s regression test yielded a p-value of 0.783 for erectile function, showing no statistically significant evidence of asymmetry in the funnel plot, suggesting that the presence of publication bias is unlikely. On the other hand, the funnel plot also showed no evidence of publication bias (Fig. 3).

### Sensitivity analysis

**Fig. 4:**
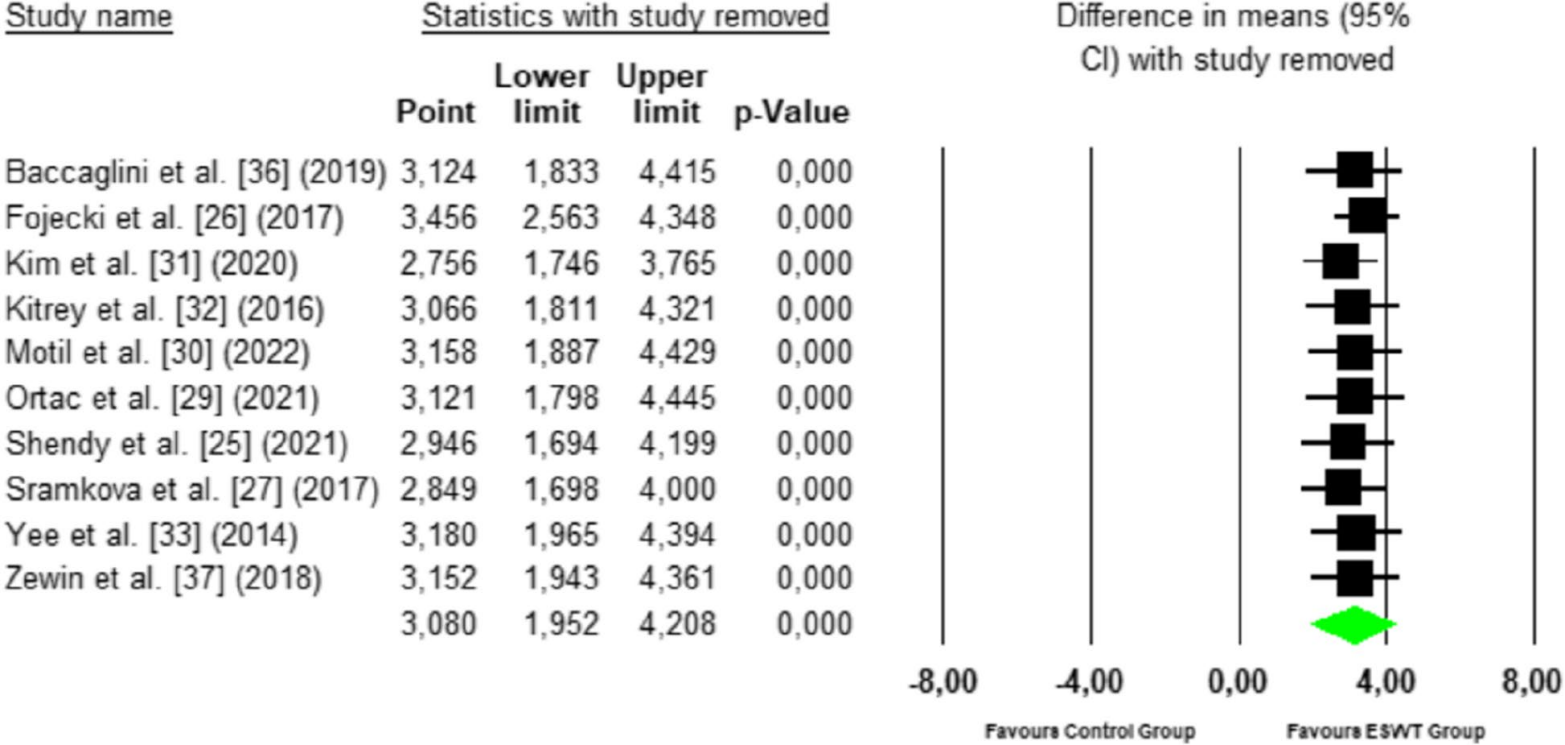
Leave-one-out plot for erectile function

The sensitivity analysis using the systematic exclusion of individual studies (Leave-One-Out) showed that the removal of any single study did not significantly alter the meta-analysis results. The overall mean difference remained stable within a range of 2.85—3.46 (95% CI: 1.70—4.42), and statistical significance was maintained in all iterations (*p* < 0.001). This suggests that the findings are robust and do not depend on a single study (Fig. 4).

Similarly, the comparison between fixed-effects and random-effects models showed a consistent effect estimate in both approaches (3.04 vs. 3.08), reinforcing the stability of the results (Fig. 2). Additionally, the subgroup analysis demonstrated the robustness of the findings, as no significant variations were observed between comparisons, further supporting the validity of the meta-analysis (Fig. 2).

## Discussion

This review aimed to evaluate the efficacy of ESWT for the treatment of ED. After the analysis of the obtained results, it could be affirmed that ESWT therapy is an effective method for the treatment of functional impotence.

Erectile function improved statistically with twelve of the interventions [23, 25, 27–32, 34–37]. Additionally, these patients achieved the Minimal Clinically Important Change Score in the erectile function domain of the International Index of Erectile Function scale [38]. In the investigations that did not identify improvements [24, 26, 33], this phenomenon could be due to the administration of an insufficient number of pulses. That is, those who did show significant improvements used an average of 26355 ± 13196 total pulses, while patients who did not show significant improvements received 6000 [26], 1500 [24] and 18000 [33]. On the other hand, Olsen et al. [24] applied a lower number of sessions than those used by the rest of the researchers (five sessions vs. nine sessions on average). Kalyvianakis et al. [34] observed a dose dependence, where the patients who received a greater number of sessions obtained better results in erectile function and penile hemodynamics. Specifically, they determined that those who received a greater number of sessions (up to 18) showed superior improvements compared to those who received 12. These findings are congruent with previous research in which ESWT has shown a dose-dependent effect, where an insufficient number of pulses or sessions did not produce the expected changes [39, 40]. However, it should also be considered that overexposure can cause tissue damage: lysis of epithelial cells, vascular damage around the treatment area, skin lesions, hematomas, petechiae… [39]. In this line, it should be noted that none of the analyzed investigations reported negative side effects. Therefore, a safe dose threshold could be 90,000 pulses [34]. Among those studies in which significant changes were obtained, four of them achieved them in a smaller number of sessions: only four [27–30]. Sramkova et al. [27], in addition to using a smaller number of sessions, also achieved these changes in a shorter experimental stage: two weeks. Furthermore, Kalyvianakis et al. [34] confirmed the maintenance of the changes experienced in the long term, after a follow-up of one year.

The quality of erection did not improve with two of the interventions analyzed [26, 33], and this effect could be attributed to the smaller number of pulses used in the treatment: 6000 [26] and 18000 [33]. Among the studies that did manage to improve this variable [24, 27, 28, 31, 32, 35, 37], Sramkova et al. [27] achieved significant improvements with a smaller number of pulses and in a shorter intervention time than the rest. Furthermore, Zewin et al. [37] demonstrated that the effects achieved were still present at nine months post-intervention.

Sexual satisfaction improved with four of the interventions [27, 31, 34, 35] but not with the one applied by Vinay [28]. The latter author carried out a protocol in which the patients received four sessions, a significantly smaller number of sessions than the other interventions, which may be one of the reasons for the lack of therapeutic efficacy. The other authors who analyzed this variable applied an average of 31,500 pulses at the end of the intervention. The effects were achieved in less time and with fewer pulses, in total, in the study by Sramkova et al. [27]. Those who performed longer-term analysis were Kalyvianakis et al. [34], confirming the initial findings after one year.

The hemodynamic changes in the cavernous arteries of the penis were significant after two of the protocols applied [25, 34]. Showing such results in a shorter intervention period, and confirming the long-term effects produced by the intervention of Kalyvianakis et al. [34], Zewin et al. [37] also included this variable in their research, although no relevant changes were found in this variable. Again, a plausible explanation for this phenomenon is the application of a smaller number of pulses in their sessions (1500 vs. 3000 pulses on average), as well as the inclusion of men with ED of surgical origin, following cytoprostatectomy. It should be noted that the improvements identified in penile blood perfusion [25, 34] could be because of neovascularization and angiogenesis resulting from the application of ESWT, as well as the immediate vasodilation associated with this intervention [13]. The energy that penetrates the tissue to be treated can generate an increase in tension in the area, as well as regenerating both blood vessels and neuronal cells. This improves erectile function by increasing blood flow and interneuronal connections [12, 13, 15].

Baccaglini et al. [36] applied an intervention in which patients, in addition to receiving ESWT, received PDEI-5. This molecule is involved in the degradation of GMPc (cyclic guanine monophosphate), producing relaxation of the smooth muscle of the corpora cavernosa and increasing the entry of blood into the corpora cavernosa to achieve erection. PDEI-5 acts by inhibiting this phosphodiesterase, increasing the concentration of GMPc in the corpus cavernosum of the penis and, consequently, its actions [41]. A long-term study was not performed to test the effect of this combined therapy, nor did the patients receive a smaller number of sessions compared to the other studies analyzed (eight vs. nine sessions on average).

The patients analyzed by Shendy et al. [25] obtained significant improvements in erectile function, where, in addition to ESWT treatment, they followed a Kegel exercise protocol. There is a direct relationship between the strength of the perineal musculature and the erectile capacity of the penis, improving erectile function through training [42]. However, in this study, no relationship was identified between combined therapy and a smaller number of sessions received or intervention time.

After the analysis of both the interventions carried out and the results obtained, it was identified that the most effective protocol for the treatment of ED by means of ESWT could be the one that used four sessions in two weeks of intervention [27], with an administration of at least 6000 pulses in each session [27], divided between the crura [25, 27, 28, 33, 34, 36] and both corpora cavernosa of the penis [24, 26, 27]. Furthermore, no additional improvements were identified by establishing weeks of rest between treatment cycles [25, 26, 31–33, 37] compared to not doing so [24, 27, 28, 34–36].

In view of the above, future lines of research suggest that new studies should be carried out to compare the effectiveness of ESWT according to the number of pulses administered, the energy flow used, and the energy penetration capacity of each of the device models used. About energy penetration, only 46.7% of the authors provide information on this parameter, ranging from 10 [26] to 70 mm [23]. Thus, progress could be made towards the protocolization and standardization of this treatment modality in ED. In addition, the most effective combination of different techniques and treatment methods, such as the administration of PDEI-5 or Kegel exercises, should also be evaluated.

### Limitations of the study

This systematic review has methodological limitations and could have extended the search period. Most of the variables analyzed by the authors were studied subjectively (questionnaires completed by the participants), thus the development and application of objective instruments would add greater validity to the results in this field of study. This systematic review also presents certain limitations related to the methodological quality of the studies. According to the risk of bias assessment (RoB tool for randomized trials), several of them exhibited a high overall risk of bias in domains such as participant blinding or the presence of incomplete data [23, 27–29, 31, 36, 37]. Similarly, in the quasi-experimental studies (evaluated using ROBINS-I), a "critical" risk of bias was identified [34, 35]. All this implies that, in a significant number of studies, factors such as non-concealed allocation, partial blinding, or participant dropouts during follow-up may be affecting the internal validity of the results. Therefore, it is recommended to interpret the findings with caution and to encourage the conduct of randomized clinical trials with greater methodological rigor and a lower risk of bias in future studies.

However, this research also presents strengths, such as the recent publication of all the articles included, and the fact that most of them are randomized controlled trials with high methodological quality. Furthermore, in comparison with other published systematic reviews, this is the one with the largest number of search terms and the largest number of databases analyzed and, consequently, it analyzes the largest number of investigations [43, 44]. Other previous reviews did include a larger number of articles in their analysis, although with fewer randomized controlled trials among their results [45–47]. For all these reasons, this systematic review, in addition to analyzing the most recent scientific evidence, is the one that provides a more reliable methodology compared to those carried out to date.

## Conclusions

The present systematic review seems to confirm the positive effect of ESWT in the treatment of ED. The application of a single treatment cycle including four sessions over two weeks of intervention and the administration of at least 6000 pulses in each session has been shown to be an effective short- and long-term schedule. In addition, the application of ESWT should be distributed throughout the crura and both corpora cavernosa of the penis.

Further studies reporting the effect of such therapy over a period longer than one year are required, as well as a standard protocol establishing the pulses, flow and energy penetration that are considered safe and effective for the resolution of ED. In any case, ESWT can be considered a therapeutic alternative to the use of drugs or vacuum erection devices.

## Data Availability

The data presented in this study are available on request from the corresponding author.

## Abbreviations

ED: Erectile dysfunction
ESWT: Extracorporeal shock waves therapy
PDEI-5: Phosphodiesterase type five inhibitor drug
